# Mean Expected Error in Prediction of Total Body Water: A True Accuracy Comparison between Bioimpedance Spectroscopy and Single Frequency Regression Equations

**DOI:** 10.1155/2015/656323

**Published:** 2015-06-02

**Authors:** Fernando Seoane, Shirin Abtahi, Farhad Abtahi, Lars Ellegård, Gudmundur Johannsson, Ingvar Bosaeus, Leigh C. Ward

**Affiliations:** ^1^Faculty of Care Science, Work Life and Social Welfare, University of Borås, 501 90 Borås, Sweden; ^2^School of Technology and Health, Royal Institute of Technology, 141 52 Huddinge, Sweden; ^3^Department of Oncology, Karolinska University Hospital, 171 76 Stockholm, Sweden; ^4^Department of Clinical Science, Intervention and Technology, Karolinska Institute, Hälsovägen 7, 141 57 Stockholm, Sweden; ^5^Department of Clinical Nutrition, The Sahlgrenska Academy, The University of Gothenburg, Sahlgrenska University Hospital, 405 30 Göteborg, Sweden; ^6^School of Chemistry and Molecular Biosciences, The University of Queensland, Brisbane, AU 7072, Australia

## Abstract

For several decades electrical bioimpedance (EBI) has been used to assess body fluid distribution and body composition. Despite the development of several different approaches for assessing total body water (TBW), it remains uncertain whether bioimpedance spectroscopic (BIS) approaches are more accurate than single frequency regression equations. The main objective of this study was to answer this question by calculating the expected accuracy of a single measurement for different EBI methods. The results of this study showed that all methods produced similarly high correlation and concordance coefficients, indicating good accuracy as a method. Even the limits of agreement produced from the Bland-Altman analysis indicated that the performance of single frequency, Sun's prediction equations, at population level was close to the performance of both BIS methods; however, when comparing the Mean Absolute Percentage Error value between the single frequency prediction equations and the BIS methods, a significant difference was obtained, indicating slightly better accuracy for the BIS methods. Despite the higher accuracy of BIS methods over 50 kHz prediction equations at both population and individual level, the magnitude of the improvement was small. Such slight improvement in accuracy of BIS methods is suggested insufficient to warrant their clinical use where the most accurate predictions of TBW are required, for example, when assessing over-fluidic status on dialysis. To reach expected errors below 4-5%, novel and individualized approaches must be developed to improve the accuracy of bioimpedance-based methods for the advent of innovative personalized health monitoring applications.

## 1. Introduction

Electrical bioimpedance (EBI) technology has advanced considerably, since the 1960s when the impedance of the body and its constituent tissues were related to an electrical current applied to the body through their water contents [1]. Subsequently, measurement of tissue and body impedance, commonly but incorrectly referred to as bioelectrical impedance analysis (BIA), was developed as practical method for assessing fat-free mass [2] and lean tissue [3] that is now in clinical use worldwide. Initial approaches for assessing body water content, and hence Fat-Free Mass (FFM), were based on impedance measurements obtained at a single frequency, typically 50 kHz (SFBIA). The impedance quotient (*H* ^2^/*Z*, or more commonly *H* ^2^/*R* where *H* is height, *Z* is impedance, and *R* is resistance) and anthropometric variables such as weight and sex were combined using regression techniques against an independent reference measurement of body water to obtain prediction equations. For the past two decades, these empirically derived prediction methods have coexisted with bioimpedance spectroscopic (BIS) methods. In BIS, impedance information is obtained from measurements acquired over a range of frequencies, typically 5 to 1000 kHz.

The BIS approach is based on the use of circuit equivalent models and Hanai mixture theory [4] with the expectation that this method would exhibit superior performance than the SFBIA methods, but to date improvements of BIS over SFBIA have been found to be marginal [5]. It would seem intuitively obvious that the larger amount of information obtained from measurements at many frequencies should provide better characterization of tissue properties and hence improve predictive power, that is, decrease expected error. In particular, BIS theory holds that impedance at a low frequency, ideally zero kHz, will be inversely related to the extracellular water (ECW) compartment volume while impedance at infinite frequency will be most closely related to total body water (TBW). However, the simplifications and assumptions invoked through the application of equivalent circuit models and the estimation of fluid volumes through mixture theory formulae reduce the value of BIS information significantly producing large variability in the obtained estimates between different EBI approaches and reference methods for the same population therefore leading to a general mistrust on EBI methods [6].

The modelling of BIS data is generally accepted to provide a good estimate of resistance at zero frequency [7] while the extrapolation of measured impedances to infinite frequency is potentially prone to measurement artefacts [8–15], overall affecting performance and accuracy of mixture theory modelling of TBW and FFM. The assumption of population mean values for several parameters in the BIS equations is an additional source of error when predicting fluid volumes in an individual. Moreover, the commonly adopted protocol of measuring whole body impedance on one side only can mask the effects of limb dominance [16] and the disproportionate contribution of limb impedance to whole body impedance [17].

Despite over 25 since the first bioimpedance-based equation for body composition analysis and all the research work and studies performed with and about bioimpedance-based prediction equations for body fluid contents, there is still very few known about the performance, besides that a good linear regression with dilution methods is shown and wide limits of agreement. It is time to learn how really well the prediction equations estimate fluid content and which methods perform the better and how much better. The specific time is now, because there is a significant upswing across dialysis wards in using EBI-based equations for guiding nephrologists to manage body fluid balance of patients requiring dialysis and such nephrologist deserve to know what is the error expected from a volume estimation based on EBI measurements.

In this study, we assessed, using the same data set and accounting for measurement artefacts, (1) the predictive advantage of BIS compared to SFBIA; (2) the predictive power of four popular 50 kHz single frequency methods compared to; (3) the performance of the two most frequently used BIS-based methods for prediction of TBW as measured by the reference method of tritium dilution.

## 2. Methods and Materials

### 2.1. Body Water Compartment Data

Comparison of methods was based upon an analysis of measurements of body water compartments performed in patients on growth hormone replacement therapy at Sahlgrenska University Hospital in Gothenburg, Sweden, between 2005 and 2011 [14]. From a complete database with 703 participants available from the aforementioned study, a total of 94 records corresponding with measurements free from high frequency artefacts have been selected; see subjects characteristics in Table 1. Data include both BIS measurements and body water measurements obtained by reference methods: TBW by tritium dilution [26], corrected for migration of tritium into nonwater compartments [27], and ECW by bromide dilution [5]. The Regional Ethics Committee of Gothenburg approved the study and all subjects gave their written informed consent for the study measurements.

### 2.2. BIS Measurements

The BIS measurements were performed on patients after at least 5 minutes in supine position, using a whole body, right-side, wrist-to-ankle (RS-WA) tetrapolar electrode configuration [28]. The impedance device used to perform the BIS measurement was the Body Scout spectrometer (Fresenius Medical Care, Germany), a predecessor of the current commercially available BCM spectrometer from the same company. All measurements were performed in the morning after a whole night of fasting. The frequency range of the BIS measurements was 5 kHz to 1 MHz. The Body Scout provides resistance (*R*) and reactance (*X*_*c*_) at each of 50 frequencies within this range, including 50 kHz. Additional parameters available included *R*_0_ and *R*_inf_ obtained from the modelling curve-fitting procedure and *T*_*d*_, a measure of the model correction [22] required to account for deviation of the data from the fitting model [29] due to measurement artefacts referred to above.

### 2.3. Artefact-Free Measurements

In order to avoid the potential influence on the study of high frequency measurement artefacts a subset of data for 94 participants was selected according to the following criteria. If the optimal fitting was obtained with a value of *T*_*d*_ close to zero, then it is expected that such BIS measurement is essentially free from measurement artefacts at high frequencies. In case of presence of measurement artefacts at high frequencies, the fitting will produce an absolute *T*_*d*_ value different to zero. The greater the magnitude of *T*_*d*_, the larger the deviation from the model. Consequently, for this analysis only BIS measurements producing *T*_*d*_ vales close to zero (±0.5) were included.

### 2.4. Single Frequency Volume Prediction Equations

Among the many SFBIA prediction equations available in the literature, four different volume prediction equations were chosen (Table 2). These equations were selected as being in wide use and have the smallest errors, best regression parameters (*R* ^2^), and low limits of agreement in cross-validation studies according to [30].

### 2.5. BIS Volume Prediction Equations

Two different approaches to the BIS method to calculate the TBW have been considered in this study. The first is the original Hanai mixture theory approach as introduced by de Lorenzo et al. in 1997 [22] and the second is a modification of this method which incorporates adjustment for subject's body mass index (BMI) as introduced by Moissl et al. [24]. In each case, the calculation of TBW is obtained as the sum of ECW + ICW; ECW and ICW are obtained with their own equations (Tables 3 and 4, resp.).

### 2.6. Statistical and Data Analysis

The agreement between the predicted values and the reference dilution volume values was assessed through both linear correlation (*r*_*p*_, Pearson) and concordance correlation (*r*_*c*_) analysis [31] with the differences between all pairs of dilution and predicted data evaluated using limits of agreement (±2SD) analysis [32] and expressed in both absolute and percentage, relative to reference TBW volume, terms. Since Bland-Altman analysis focuses on agreement and not on accuracy, the absolute percentage difference obtained from every prediction was determined and plotted in a diagram combining a distribution plot with a scatter plot, similar to Bland-Altman. This allowed the Mean Absolute Percentage Error (MAPE) in % [33] for all the predictions performed with each different method to be computed.

**Figure 1:**
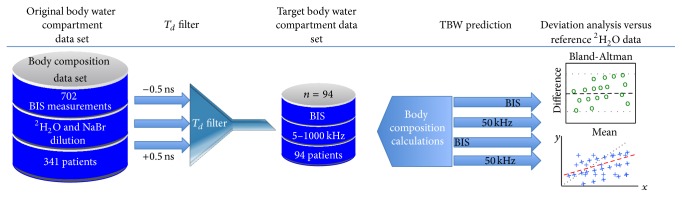
Flow chart of sequence of analytical steps.

All the calculations for volume prediction [31], statistical analysis, and comparison performance including plots were performed with MATLAB 8.1 (The MathWorks Inc., MA, USA). The sequence of analytical steps applied in this study for evaluating performance differences between 50 kHz single frequency methods and BIS methods is described in the flow chart of Figure 1.

## 3. Results

### 3.1. Effect of Measurement Frequency on Correlation between Impedance and Body Water Volumes

**Figure 2:**
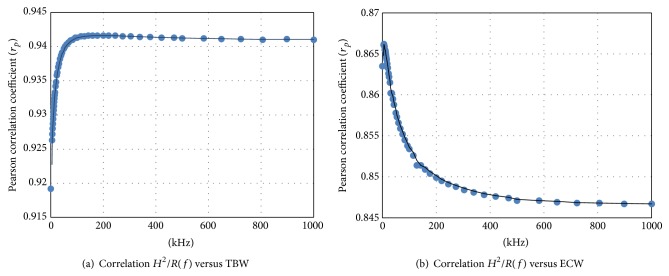
Correlation of impedance quotients *H* ^2^/*R* (kHz) and estimated body fluids at different frequencies. Total body water in panel (a) and extracellular fluid in panel (b).

The correlation between the impedance quotient, *H* ^2^/*R*, and TBW or ECW, at each of the measured frequencies for all subjects that had complete data (*n* = 607) is presented in Figures 2(a) and 2(b), respectively. Correlations (*r*_*p*_) for TBW were high (>0.919 at 0 kHz) rising to a maximum of 0.942 at approximately 143 kHz and then declining progressively to 0.941 at infinite frequency. The opposite pattern was seen for ECW with maximum correlation of 0.866 at 5 kHz declining progressively to 0.846 at infinite frequency. Notably, the correlation at zero frequency, 0.863, was lower than that at measured frequencies of 20 kHz or lower.

### 3.2. Correlation between Predicted and Measured TBW

**Figure 3:**
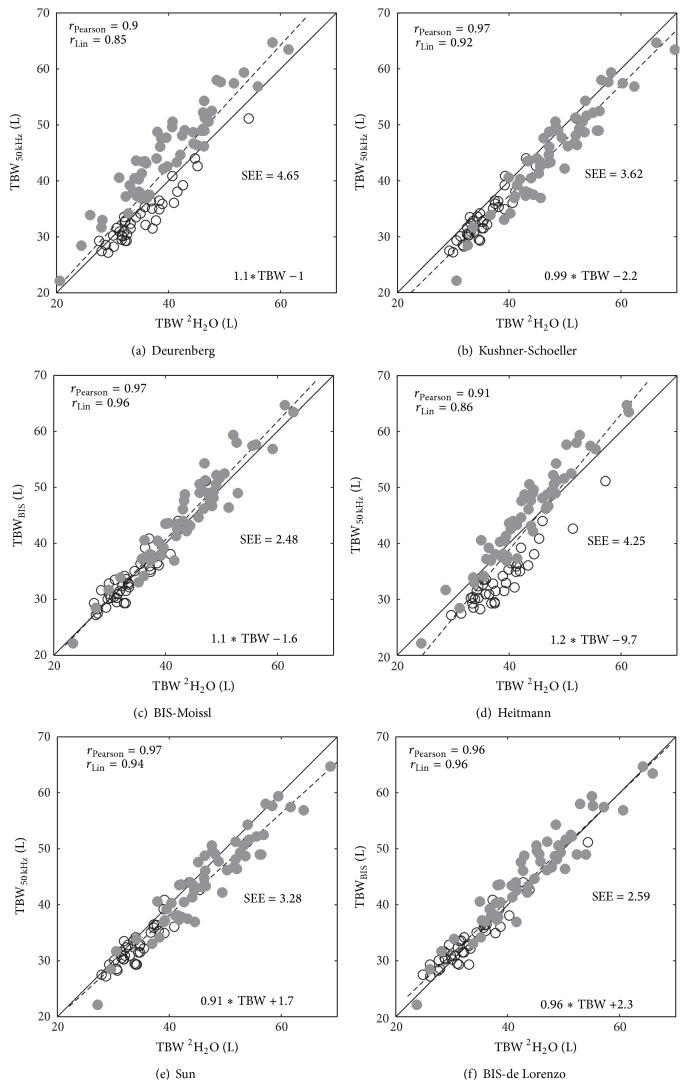
Comparison of TBW volume predicted by impedance methods with TBW measured by tritium dilution. Male data plotted with solid circle and female data with hollow circle.

The correlations between predicted TBW and measured TBW volumes based on tritium dilution are presented in Figure 3. All impedance methods were highly and significantly correlated (*r*_*c*_ > 0.9, *P* < 0.0001) with the reference method although the strength of the correlation varied between prediction methods, varying from 0.90 for the Deurenberg [34] equation to 0.97 for the BIS method of Moissl et al. [24] (Figures 3(a) and 3(e), resp.). Similarly the SEE varied between methods: the lowest values, 2.48 L and 2.59 L, being observed for the two BIS methods of [24] and de Lorenzo et al. [22], respectively.

### 3.3. Limits of Agreement between Predicted and Measured TBW

**Figure 4:**
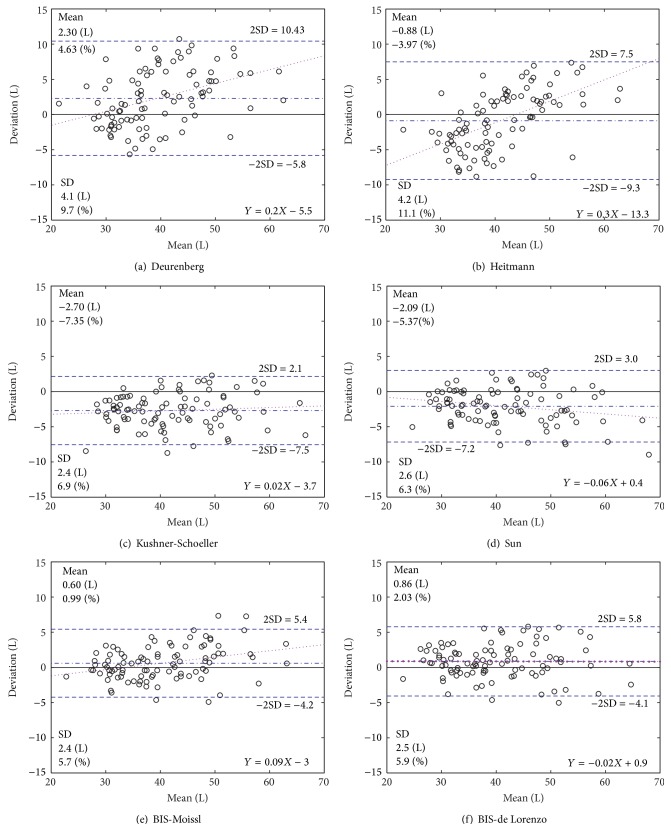
Limits of agreement between predicted TBW volume and TBW volume determined by tritium dilution; data points are shown by circle while mean ± 2SD limits of agreement and fitted regression line to the data are depicted by dash/dot lines, respectively. The equation of the fitted regression equation, SEE, Pearson correlation coefficient, and mean TBW volume (L and %) are also shown. The values for the limits of agreement are indicated below or under the corresponding line.

Limits of agreement between methods are plotted in Figure 4. The smallest mean biases were observed for the BIS and Heitmann predictors, 0.60, 0.86, and 0.88 L according to the Bland-Altman plots in Figures 4(b), 4(e), and 4(f), respectively. The smallest 2SD limits of agreement were obtained for the BIS methods and the SFBIA prediction of Kushner (Figures 4(c), 4(e), and 4(f)): the SD for these comparisons being 2.4 L (5.7%), 2.5 L (5.9%), and 2.4 L (6.9%), respectively.

Most notably the errors in predictions with the 50 kHz methods of Deurenberg and Heitmann (Figures 4(a) and 4(b)) were positively and significantly dependent upon the magnitude of TBW volume (*r*_*p*_ = 0.5767, *P* < 0.001 for Deurenberg, and *r*_*p*_ = 0.71, *P* < 0.001 for Heitmann).

The standard deviation (SD) for each of the 50 kHz single frequency methods (6.9% and 6.3% for Kushner et al. and Sun et al., resp.; Figure 4 and Table 5) was both similar but slightly larger than the standard deviations obtained for the BIS-based methods of 5.7% and 5.9% for Moissl et al. and de Lorenzo et al., respectively. The mean of the differences in the Bland-Altman plots, that is, the bias, was, however, markedly smaller: 0.99% for the BIS-based approach of Moissl and 2.03% for de Lorenzo compared with −5.37% and −7.35% for Sun et al. and Kushner-Schoeller, respectively. The magnitudes of the biases were similar when the population was stratified by sex (Table 6). Notably, the biases were larger for males than females despite there being more males than females (56 : 38).

### 3.4. Performance of Predictors for Accuracy of the Estimations

Table 7 presents data assessing the performance of the methods when producing a single estimation, that is, the expected error for any given estimation based in a single BIS measurement. The values indicate that it is expected that none of the approaches will produce an estimate closer to the tritium-dilution value than 1.16% for women and 1.49% for men in any case. Both BIS-based methods exhibit better performance than the 50 kHz single frequency methods that have deviation values in the range typically around 5%. Overall, predictions produced by the Sun equation, which, although still producing an error larger than the BIS methods, provides estimates closest to the reference dilution values.

**Figure 5:**
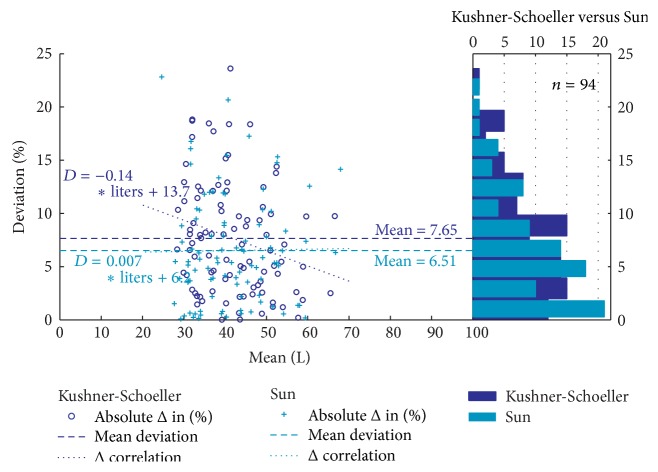
Relative deviation Bland-Altman plots combined with distribution plot for the prediction of TBW volume obtained from ^2^H_2_O dilution and 50 kHz single frequency bioimpedance regression equations.

Figure 5 presents the absolute difference as percentages on a distribution plot combined with a scatter plot. The scatter plot shows the value for the absolute differences in percentages rather than the differences in litres as in limits of agreement plot. It is clear, reinforcing the data in Table 7, that the Sun prediction equation, as judged by smaller values of the MAPE and confidence intervals, produces slightly better estimation of TBW than other single frequency methods.

### 3.5. Comparison of Different BIS Methods

**Figure 6:**
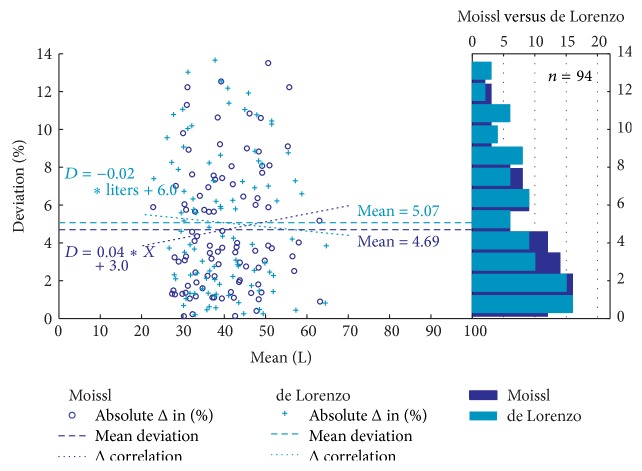
Relative deviation Bland-Altman plots combined with distribution plot for the prediction of TBW volume obtained from tritium dilution and BIS prediction equations.

The two BIS methods produced similar values for the mean of the absolute deviation when compared to the reference method, 4.69% and 5.07% for Moissl and de Lorenzo, respectively, as shown in the combined plot in Figure 6. Both the mean of MAPE and the SD limits of agreement were similar (Table 7) for both men and women. Both methods showed a very slight dependency of the deviation with absolute TBW volume but in opposite directions; in both cases the deviation was not significant: Moissl (*r*_*p*_ = 0.14); de Lorenzo (*r*_*p*_ = 0.007).

## 4. Discussion

This study has produced a number of key findings. Firstly, despite the commonly held view that on theoretical grounds, BIS should provide significantly better predictions of body fluid volumes than SFBIA methods; this is not necessarily the case. It is clear that measurement of impedance at a low frequency is most closely correlated with ECW volume although not the theoretically optimal frequency of zero frequency. The slightly poorer correlation at zero frequency is most likely due to error induced by extrapolation of measured data to the practically immeasurable zero frequency. It appears that, operationally, ECW can be well predicted at any frequency below approximately 20 kHz. This concurs with previous observations using BIS to measure ECW in lymphedema [35, 36].

Secondly, TBW is most closely correlated with impedance at high frequencies as theory would predict. Again, extrapolation of measured data to the theoretically optimal frequency for prediction of TBW, infinite frequency, slightly worsened the prediction. The difference between the maximum observed correlation, at 143 kHz, and that at infinite frequency was, however, only 0.001 units. The data suggest that TBW may be equally well predicted from impedance measured at any high frequency above approximately 140 kHz.

The third notable finding of the present study is that, despite the above observations, BIS methods in practice have better predictive performance than the single frequency equations as judged by smaller biases, limit of agreement, and absolute error of the four SFBIA equations tested. These three findings suggest that prediction of fluid volumes by SFBIA is markedly influenced by the inclusion of predictor variables other than the impedance values themselves in the prediction algorithm. This supports previous observations from a number of studies [5, 37] that inclusion of impedance in prediction equations, whilst improving overall predictive power, is adding to the already powerful predictive value of variables such as weight, height, age, and sex.

The four SFBIA equations all perform slightly but significantly different to each other. The Pearson correlation and the Lin concordance coefficients range from 0.9 and 0.86 to 0.97 and 0.94, respectively, with the Kushner-Schoeller and Sun predictors performing better than Deurenberg and Heitmann exhibiting smaller biases, limits of agreement, and MAPE values. Kushner-Schoeller and Sun exhibited similar performances as judged by correlation (*r*_*p*_ = 0.97 for both; *r*_*c*_ = 0.92 and *r*_*c*_ = 0.94, resp.) but Sun's prediction equations produced better agreement and also smaller MAPE. Interestingly, the two worse performing predictors, Heitmann and Deurenberg, both included variables other than the impedance quotient and weight raising the possibility that inclusion of additional predictor variables while improving prediction within that study population decreases the portability of predictors between populations. For example, Deurenberg's prediction equation showed poor performance in this study with low values of correlation, high SEE, large agreement bias, and limits of agreement, but the same equation when cross-validated in the original study [34] produced a correlation of *r*_*p*_ = 0.95 and a SEE of 1.95 L.

A possible explanation for the significantly better performance indicators with Sun's equation than with the other SFBIA methods may lie with the larger number of subjects used in that study, 1035 women and 734 men, compared to the others, for example, 40 subjects only for Kushner-Schoeller.

The conclusions from this analysis could be criticised as being relevant only to these particular SFBIA equations. The equations used here were chosen because of their popularity, they still in use 20–25 years after being formulated [38, 39], and as being representative of the many SFBIA equations that have been published, see [40]. Although all equations perform differently, when applied to broadly similar populations, the magnitude of interequation differences is generally small and similar to that observed here. Of more importance are their overall accuracy and their performance relative to that of BIS techniques. Despite high correlations (*r*_*p*_ = 0.97 and *r*_*c*_ = 0.94) and relatively small limits of agreement when compared with the reference method of tritium dilution (SD = 2.6 L) and low deviation for single estimations of TBW, particularly in women (MAPE of 5.76%), the overall performance of SFBIA cannot be considered equivalent to the performance of the BIS prediction equations.

The better performance exhibited by BIS prediction equations supports the original contention [41] that the BIS approach had the potential to improve predictive power. To date, however, this had not generally proven the case [42]. Despite the better performance of BIS methods seen in the present study, the mean bias when estimating TBW was approximately 5% or around 2 litres, values in agreement with those presented by Moon et al. in [43]. This level of error may be acceptable in some clinical settings where measures of body composition are required, for example, in weight control, but is unlikely to be acceptable where TBW is being measured in a clinically critical setting such as renal failure. It is important to recognize that consideration of mean bias may obscure much larger prediction errors for some individuals. In 40 of the 94 subjects BIS-based predictions produced larger deviations than the mean with 20 out 94 being greater than 7.5% or approximately 3 litres. Unfortunately, it is not possible* a priori* to identify which individuals fall at the extremes of the error distribution. Such uncertainty casts a shadow of unreliability over BIS and impedance prediction of body composition in general that is difficult to overcome.

Clearly, efforts are required to improve the predictive performance of BIS methods. The attempt by Moissl et al. [24] to combine several equation parameters into a single coefficient indexed to individual subject BMI was an important improvement to the original mixture theory approach [22]. This approach attempted to account for anthropometric differences between subjects with different body masses and geometry. Unfortunately, as this study has demonstrated, improvements in predictive performance were marginal. The fundamental problem is that, as also indicated in [42, 44], BIS equations inherently require simplifications and assumptions based on population mean values as being accurately applicable to all subjects. This way is producing an acceptable performance at population level but producing completely unacceptable errors at measurement level as recently pointed by Piccoli in [44]. Therefore, the selection of values for some intrinsic parameter of the BIS prediction equation must be modified in such a manner that they are more personalized. Most probably such personalization will be required not only at individual subject level but also for each pathological or disease state that may influence body water volumes or the conductivity of body fluids.

## 5. Conclusions

From one side, it is shown that BIS equations can predict with better accuracy TBW, but the exhibit expected error might not be sufficiently small to justify its use in clinical application where the accuracy of the estimation must be below 1 L (approximated 2,5%) like in dialysis [45]. The dialysis case is extremely important and despite the reported poor accuracy and other reported limitations [46], the use of BIS is spreading among dialysis clinics and requires immediate targeted actions to improve its clinical usefulness.

In addition, as indicated recently in [6], if the accuracy is refined there are clinical populations other than dialysis patients that could potentially benefit from the advantages associated with bioimpedance technology, for example,* noninvasiveness*,* safety*,* ease of use*,* portability*, and* relatively low cost medical technology*.

## Figures and Tables

**Table 1:** Number of patients, average values for the anthropometric variables, and volume estimation for TBW (mean ± SD).

Number of patients		Age	Height	Weight	BMI	*T*_*d*_	TBW
Male	Female	(Years)	(cm)	(Kg)	(Kg/m^2^)	(s ×10^−9^)	(L)
56	38	61 ± 14	172 ± 10	84.9 ± 16.9	28.7 ± 5.07	0.29 ± 0.14	40.47 ± 9.2

Note: BMI: body mass index; TBW: total body water; *T*_*d*_: time delay of Cole data modelling.

**Table 2:** 50 kHz single frequency prediction equations.

Source	Equation
Deurenberg et al. [18]	6.69 + 0.34573*H* ^2^/*Z*_100_ + 0.17065 weight − 0.11 age + 2.66 sex

Heitmann [19]	−17.58 + 0.240*H* ^2^/*R*_50_ − 0.172 weight + 0.40 sex weight + 0.165 height

Kushner and Schoeller [20]	Men = 8.399 + 0.396*H* ^2^/*R*_50_ + 0.143 weight Women = 8.315 + 0.382*H* ^2^/*R*_50_ + 0.105 weight

Sun et al. [21]	Men = 1.203 + 0.499*H* ^2^/*R*_50_ + 0.176 weight Women = 3.747 + 0.450*H* ^2^/*R*_50_ + 0.113 weight

**Table 3:** BIS volume prediction equations using de Lorenzo's method [22].

Body fluid	Equation	Variable values
ECW	$\frac{1}{1000}{\left(\frac{{K_{b}}^{2}{\rho_{e}}^{2}}{D_{b}}\right)}^{1/3}{\left(\frac{\sqrt{W}H^{2}}{R_{e}}\right)}^{2/3}$	*K*_*b*_ = 4.3 *D*_*b*_ = 1.05 × 10^−3^ *ρ*_*e*_ = 40.5 for men

ICW	${\left(1+\frac{\text{ICW}}{\text{ECW}}\right)}^{5/2}=\left(\frac{R_{e}+R_{i}}{R_{i}}\right)\left(1+\frac{\rho_{i}}{\rho_{e}}\frac{\text{ICW}}{\text{ECW}}\right)$	*ρ*_*e*_ = 39 women *ρ*_*i*_ = 273.9 men *ρ*_*i*_ = 264.9 women

Note: ICF is obtained after solving the resulting 5th grade equation after substituting *x* = ICW/ECW according to [23].

**Table 4:** BIS volume prediction equations using BMI compensation.

Body fluid	Equation	*K*_*te*_ values
ECW	${K_{ef}\left(\frac{{\text{height}}^{2}\cdot \sqrt{\text{weight}}}{R_{e}}\right)}^{2/3}$	*K*_*ef*_ = 0.188/BMI + 0.2883

ICW	${K_{if}\left(\frac{{\text{height}}^{2}\cdot \sqrt{\text{weight}}}{R_{i}}\right)}^{2/3}$	*K*_*if*_ = 5.8758/BMI + 0.4194

Note: *K*_*ef*_ and *K*_*if*_ were obtained by regression to minimize the dependency of the error from BMI in [24, 25].

**Table 5:** Bland-Altman analysis for comparison of total body water predicted by BIS and 50 kHz impedance methods.

Prediction method	Volume (L)	Bias^1^		Correlation		SEE
		Liters (mean ± SD)	% (mean ± SD)	Pearson	Lin	
Reference method ^2^H_2_O dilution	40.47 ± 9.2					

Deurenberg et al. [18]	38.6 ± 7.7	2.3 ± 4.1	4.63 ± 9.7	0.90	0.85	4.65
Heitmann [19]	41.35 ± 6.9	−0.88 ± 4.2	−3.97 ± 11.1	0.90	0.86	4.25
Kushner and Schoeller [20]	43.17 ± 9.0	−2.70 ± 2.4	−7.35 ± 6.9	0.97	0.92	3.62
Sun et al. [21]	42.56 ± 9.7	−2.09 ± 2.6	−5.37 ± 6.3	0.97	0.94	3.28

BIS prediction methods						
Moissl et al. [24]	39.9 ± 8.5	0.60 ± 2.4	−0.99 ± 5.7	0.97	0.96	2.48
de Lorenzo et al. [22]	39.6 ± 9.22	0.86 ± 2.5	−2.03 ± 5.9	0.96	0.96	2.59

^1^Compared to reference method.

**Table 6:** Total body water predicted by different impedance methods according to sex.

Prediction method	Total body water			
	Women		Men	
	Volume (L)	Bias (L, %)	Volume (L)	Bias (L, %)
Reference method^ 2^H_2_O dilution	33.59 ± 4.98		45.14 ± 8.52	

50 kHz prediction methods				
Deurenberg et al. [18]	35.24 ± 5.56	(+1.65, 4.91)	40.15 ± 8.27	(−4.99, −11.07)
Heitmann [19]	38.60 ± 5.46	(+5.01, 14.92)	43.22 ± 7.18	(−1.92, −4.25)
Kushner and Schoeller [20]	35.79 ± 4.46	(+2.2, 6.55)	48.18 ± 7.82	(+3.04, 6.71)
Sun et al. [21]	35.25 ± 5.11	(+1.66, 4.94)	47.53 ± 9.09	(+2.39, 5.27)

BIS prediction methods				
Moissl et al. [24]	33.62 ± 4.61	(+0.03, 0.09)	44.11 ± 7.85	(−1.0, −2.21)
de Lorenzo et al. [22]	33.26 ± 5.72	(−0.33, −0.98)	43.92 ± 8.71	(−1.23, −2.72)

**Table 7:** Mean absolute deviation predicting TBW: evaluation of accuracy.

%		EBI modality	Method	Liters	
Women (mean ± SD)	Men (mean ± SD)			Women (mean ± SD)	Men (mean ± SD)
4.47 ± 3.3	4.85 ± 3.3	BIS	Moissl et al. [24]	1.53 ± 1.2	2.22 ± 1.7
4.63 ± 3.3	5.36 ± 3.9	BIS	de Lorenzo et al. [22]	1.54 ± 1.1	2.39 ± 1.8
5.79 ± 4.3	11.13 ± 5.9	50 kHZ	Deurenberg et al. [18]	1.93 ± 1.4	5.00 ± 2.8
15.18 ± 6.4	5.88 ± 3.7	50 kHZ	Heitmann [19]	5.01 ± 2.0	2.69 ± 1.9
7.37 ± 4.9	8.39 ± 6.9	50 kHZ	Kushner and Schoeller [20]	2.36 ± 4.5	3.44 ± 2.7
5.76 ± 4.6	7.02 ± 5.3	50 kHZ	Sun et al. [21]	1.90 ± 1.5	3.06 ± 2.2
